# Huge variability in restrictions of mobilization for patients with aneurysmal subarachnoid hemorrhage - A European survey of practice

**DOI:** 10.1016/j.bas.2023.101731

**Published:** 2023-03-21

**Authors:** Iftakher Hossain, Alexander Younsi, Ana Maria Castaño Leon, Laura Lippa, Péter Tóth, Nicole Terpolilli, Lovisa Tobieson, Francesco Latini, Andreas Raabe, Bart Depreitere, Elham Rostami, Trauma & Critical Care section of the European Association of Neurosurgical Societies

**Affiliations:** a Neurocenter, Department of Neurosurgery, Turku University Hospital, Turku, Finland; b Department of Clinical Neurosciences, Neurosurgery Unit, University of Cambridge, Addenbrooke's Hospital, Cambridge, United Kingdom; c Department of Neurosurgery, University Hospital Heidelberg, Heidelberg, Germany; d Department of Neurosurgery, Hospital Universitario 12 de Octubre, Instituto de Investigación Sanitaria Hospital 12 de Octubre (imas12), Madrid, Spain; e Department of Neurosurgery, Ospedale Niguarda, Milano, Italy; f Department of Neurosurgery, University of Pecs, Hungary; g Department of Neurosurgery, Munich University Hospital, Munich, Germany; h Department of Neurosurgery of Linköping, Linköping University, Linköping, Sweden; i Department of Biomedical and Clinical Sciences, Linköping University, Linköping, Sweden; j Department of Medical Sciences, Section of Neurosurgery, Uppsala University, Uppsala, Sweden; k Department of Neurosurgery, Inselspital, Bern University Hospital, University of Bern, Bern, Switzerland; l Department of Neurosurgery, University Hospitals Leuven, Leuven, Belgium; m Department of Neuroscience, Karolinska Institute, Stockholm, Sweden

**Keywords:** Subarachnoid hemorrhage, Early mobilization, Head-of-bed elevation, Cerebral vasospasm

## Abstract

**Introduction:**

One of the major goals of neurointensive care is to prevent secondary injuries following aSAH. Bed rest and patient immobilization are practiced in order to decrease the risk of DCI.

**Research question:**

To explore the current practices in place concerning the management of patients with aSAH, specifically, protocols and habits regarding restrictions of mobilization and HOB positioning.

**Material and methods:**

A survey was designed, modified, and approved by the panel of the Trauma & Critical Care section of the EANS to cover the practice of restrictions of patient mobilization and HOB positioning in patients with aSAH.

**Results:**

Twenty-nine physicians from 17 countries completed the questionnaire. The majority (79.3%) stated that non-secured aneurysm and the presence of an EVD were the factors related to the establishment of restriction of mobilization. The average duration of the restriction varied widely ranging between 1 and 21 days. The presence of an EVD (13.8%) was found to be the main reason to recommend restriction of HOB elevation. The average duration of restriction of HOB positioning ranged between 3 and 14 days. Rebleeding or complications related to CSF over-drainage were found to be related to these restrictions.

**Discussion and conclusion:**

Restriction of patient mobilization regimens vary widely in Europe. Current limited evidence does not support an increased risk of DCI rather the early mobilization might be beneficial. Large prospective studies and/or the initiative of a RCT are needed to understand the significance of early mobilization on the outcome of patients with aSAH.

## Highlights

• Average duration of restriction of mobilization varied widely between 1 and 21 days.
• Average duration of restriction of HOB positioning ranged between 3 and 14 days.
• Non-secured aneurysms and EVD, main reasons for recommending restricted mobilization.
• Large prospective studies and/or RCTs are needed.

## 1 Introduction

Aneurysmal subarachnoid hemorrhage (aSAH) remains a disastrous event with an initial mortality estimated at about 15%, rising to 40% within one month after the insult (Ingall et al., 2000). Although acute management and rehabilitation of this rare but impactful form of hemorrhagic stroke have been advanced through continuous, interdisciplinary efforts, resulting in an improved survival rate in the last decades (Nieuwkamp et al., 2009), affected patients still suffer from long-term physical and cognitive impairments in up to 50% of cases (Hop et al., 1998). Accordingly, lifetime costs to care for aSAH patients are high and far exceed those of ischemic stroke patients (Taylor et al., 1996).

Recovery after aSAH is typically complicated by intracranial sequelae such as delayed rebleeding, seizure, delayed cerebral ischemia (DCI) or hydrocephalus, mostly requiring drug treatments, surgical interventions and prolonged monitoring in an intensive care unit (ICU) (Diringer, 2009). In addition, immobility and critical illness, often stemming from extracranial complications such as cardiac dysfunction or electrolyte disorders (Levine, 2008), put the patients at risk for developing further pulmonary, cardiovascular, and neuromuscular deficiencies (Al-Khindi et al., 2010; Friedman et al., 2003; Ray et al., 2009). Before securing the aneurysm, aSAH patients are typically treated with prescribed bed rest and immobility, aiming at the reduction of possible rebleeding. In addition, the most feared complications that can occur days following bleeding are DCI and delayed neurological dysfunction (DND) and the hemodynamic changes are believed to be a major contributing factor. Thus, restriction in patient mobilization such as bed rest or head-of-bed (HOB) positioning has been practiced even after effective aneurysm treatment in order to maintain adequate cerebral blood flow (Mendez-Tellez et al., 2012). However, in absence of specific recommendations and any class one evidence regarding early mobilization, in current guidelines, marked practice variation might exist (Connolly et al., 2012; Steiner et al., 2013; Hernandez et al., 2018).

This contrasts with current “early mobilization programs” for patients with critical illness and mechanical ventilation, typically consisting of a multidisciplinary approach to increase their participation in upright functional activity (Lai et al., 2017). Such early mobilization has proven to be safe, helped to reduce ICU- and hospital length of stay as well as complications associated with, respiratory complications, critical illness and even improved functional outcomes (Needham, 2008; Schweickert et al., 2009; Wang et al., 2020). Of note, the effect of physical therapy in the ICU on intracranial pressure (ICP) and cerebral perfusion pressure (CPP) have already been assessed for e.g., passive range of motion and active exercise, and no relevant elevation was found (Brimioulle et al., 1997; Koch et al., 1996). Similarly, positional changes in patients with aSAH such as HOB elevation did not alter cerebral hemodynamics or increase the incidence of DCI (Zhang and Rabinstein, 2011; Blissitt et al., 2006). Accordingly, the concept of early mobilization has been generally accepted in other acute neurological disorders such as stroke, intracranial hemorrhage (ICH) or traumatic brain injury (TBI). (Diserens et al., 2012; Yen et al., 2020; Andelic et al., 2012).

The aim of current study was to assess the existing practice variations at different neurosurgical care providers in Europe as well as to investigate if there is any evidence of the benefit of restriction in patient mobilization.

## 2 Methods

### 2.1 Development and approval

At first, a group of members of the Trauma & Critical Care section of the European Association of Neurological Surgeons (EANS), representing eight different neurosurgical centers, discussed their local practise of mobilization/HOB positioning after aSAH in detail. Next a survey was designed to cover the practice of restriction of patient mobilization and HOB positioning at each center (Appendix A). The questionnaire was piloted and modified based on feedback by members of the writing group of this paper. The Trauma & Critical Care section of the EANS approved the survey content and its subsequent dissemination. The questionnaire consists of eight items, including two questions regarding country, city, and name of the hospital at which the respondent is based. The questionnaire was built into Google Forms® and sent digitally to in-charge neurosurgeons that were contacted by email.

### 2.2 Dissemination

We disseminated the questionnaire to the Trauma & Critical Care and Vascular sections of the EANS in a collaborative effort to assess variation in practice.

### 2.3 Analysis

Results of the survey were analyzed using descriptive statistics. Categorical variables are presented as numbers and frequencies, unless stated otherwise. Differences in the practice of restriction of mobilization or HOB positioning among countries were not evaluated.

## 3 Results

### 3.1 General information

29 physicians from 17 countries completed the questionnaire. Among countries, Germany and Sweden had the highest rate of answers (four physicians) followed by Spain, Switzerland, UK, Greece, Hungary, and the Netherlands (two physicians each). Except for one physician that was based in Singapore but previously worked in UK, all the respondents were based in European countries. Apart from the Hospital Universitario 12 de Octubre and the University of Pecs Clinical center, the practice of an individual center was reflected by only one physician response.

Except for three respondents (10.3%) based in UK, Portugal and Sweden, the remainder of respondents (89.7%) confirmed the use of a specific protocol for the management of aSAH patients.

General information from the survey results and respondents is described in Table 1.

**Table 1:** Repressents general information from the survey results and respondents.

Country	Number of centers	SAH protocol	Restriction of mobilization			Restriction of HOB positioning		
			Aneurysmal SAH (aSAH)	Idiopathic non-aneurysmal SAH (iSAH)	Duration	Aneurysmal SAH (aSAH)	Idiopathic non-aneurysmal SAH (iSAH)	Duration
**Austria**	1	Yes	If non secured aneurysm or EVD	EVD	7–10 days	Never	Never	
**Belgium**	1	Yes	Never	Never		Never	Never	
**Czech Republic**	1	Yes	If non secured aneurysm or EVD	Never	2–5 days	EVD	Never	3–7 days
**Germany**	4	Yes	All cases	All cases	14–21 days for aHSA	Never	Never	
					3–5 days for iSAH			
		Yes	If non secured aneurysm	Never	1 day	Never	Never	
		Yes	Never	Never		Never	Never	
		Yes	If secured aneurysm	Never	14 days	If secured aneurysm	Never	14 days
**Greece**	2	Yes	If non secured aneurysm or VD	EVD	7 days	If non secured aneurysm	Never	7 days
		Yes	If non secured aneurysm or EVD	EVD	14–21 days for non-secured aneurysm	EVD	EVD	Until EVD is removed
					7 days for secured aneurysm with EVD			
					3–5 days for iSAH			
**Hungary**	2	Yes	All cases	Never	7–10 days	All cases	Never	7–10 days
		Yes	All cases	Never	7–10 days	All cases	Never	7–10 days
**Italy**	1	Yes	If non secured aneurysm or EVD	All cases	10–14 days	Never	Never	
**Lithuania**	1	Yes	If non secured aneurysm	Never	3–5 days	EVD	EVD	Until EVD is removed
**Netherlands**	2	Yes	If non secured aneurysm	Never	Not state	EVD	EVD	Not state
		Yes	EVD	EVD	Until EVD is removed	If non secured aneurysm or EVD	Never	Until aneurysm is secured or EVD removed
**Poland**	1	Yes	All cases	All cases	7–10 days	Never	Never	
**Portugal**	1	No	If non secured aneurysm	Never	1 day	Never	Never	
**Russia**	1	Yes	If non secured aneurysm or EVD	EVD	7–10 days	EVD	EVD	7–10 days
**Singapore**	1	Yes	EVD	EVD	Until neurological stable to assess EVD closure	Never	Never	
**Spain**	2	Yes	Never	Never		Never	Never	
		Yes	Never	Never		Never	Never	
**Sweden**	4	No	If non secured aneurysm	Never	1–2 days	Never	Never	
		Yes	If non secured aneurysm	Never	1 day	Never	Never	
		Yes	All cases	Never	1 day after aneurysm is secured	All cases	Never	Not described
		Yes	All cases	Never	10 days	All cases	Never	10 days
**Switzerland**	2	Yes	All cases	Never	10–14 days	If secured aneurysm		10–14 days
		Yes	All cases	Never	10–14 days	If secured aneurysm		10–14 days
**UK**	2	No	Never	Never		Never	Never	
		Yes	If non secured aneurysm	Never	1–3 days	Never	Never	

SAH ​= ​subarachnoid hemorrhage. HOB ​= ​head-of-bed.

### 3.2 Restriction of mobilization

**Fig. 1:**
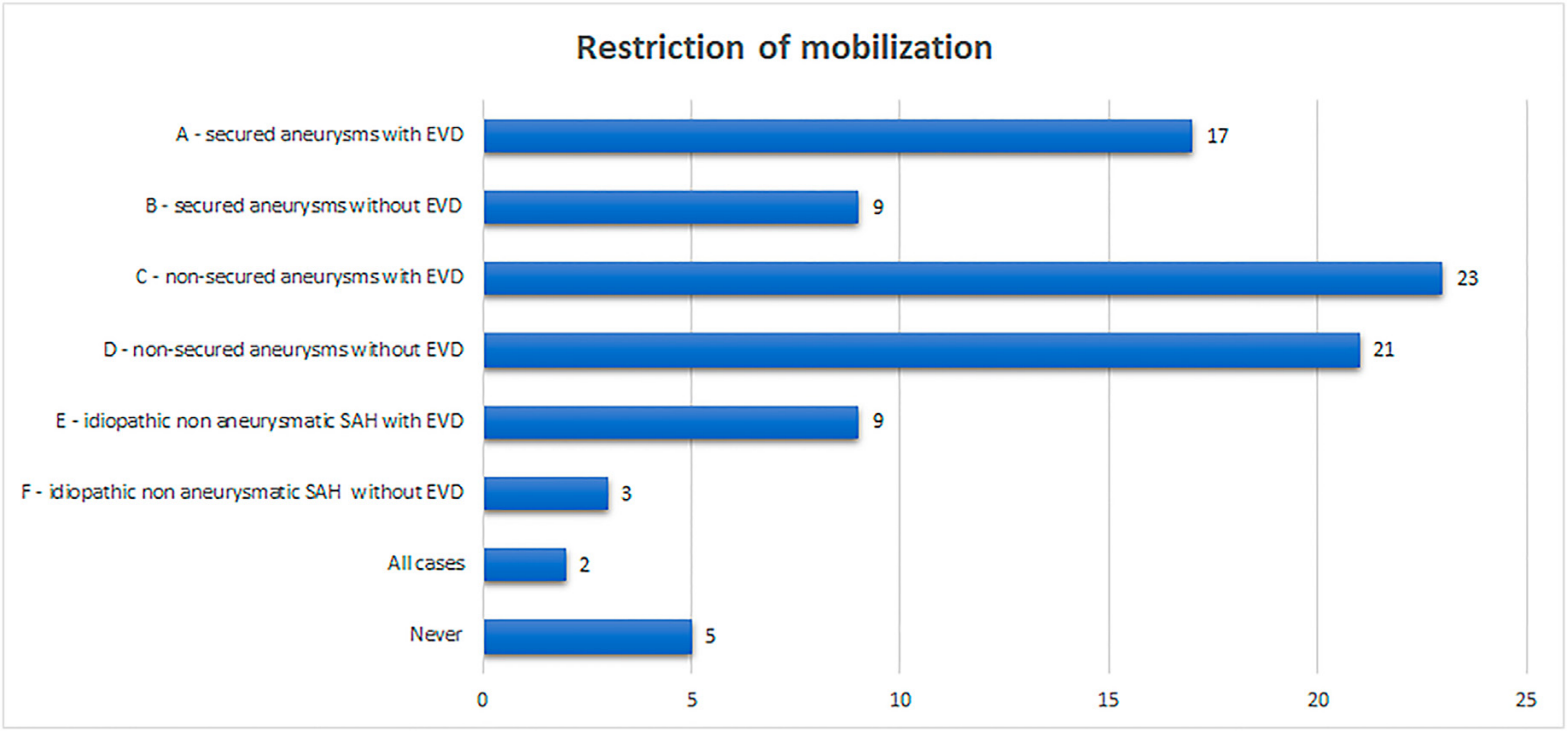
Results of responses where the responders could select more than one answer regarding the recommendation of restriction of mobilization between different scenarios.

Respondents could select more than one answer for question 4, which led to noticeable differences in the recommendation of restriction of mobilization between different scenarios (Fig. 1).

The most frequent combination of answers was to recommend restriction of mobilization for non-secured aneurysms regardless of the presence of an EVD. This answer was stated by seven respondents (24.1%). The second most frequent combination of answers was to extend the previous recommendation to all patients with aSAH (six respondents, 20.7%) irrespective of the status of the aneurysm or the need of an EVD. Four (13.8%) respondents recommended restriction of mobilization in two different scenarios: the aneurysm is not secured or for those patients with SAH of any origin with EVD. Two respondents (6.9%) recommended restriction of mobilization for all patients with EVD. Five (17.2%) respondents would never state that kind of recommendation for any SAH patient whereas two respondents (6.9%) would recommend restriction of mobilization in all cases of SAH.

#### 3.2.1 Secured aneurysms

For secured aneurysms, 17 out of 29 (58.6%) respondents would recommend restriction of mobilization for those patients that required EVD placement. However, for patients with a secured aneurysm although not having an EVD, the same restriction would be recommended according to the answers of nine (31%) respondents.

#### 3.2.2 Non-secured aneurysms

For non-secured aneurysms, the rate of recommendation to restrict mobilization after SAH increased to 23 (79.3%) and 21 (72.4%) respondents, respectively, depending on the presence or absence of an EVD.

#### 3.2.3 Idiopathic non-aneurysmatic SAH

For idiopathic non-aneurysmatic SAH, the rate of recommended restriction of mobilization was lower compared to patients with aSAH. For patients that required an EVD, 9 (31%) respondents would recommend restriction of mobilization. Nevertheless, three respondents (10.3%) would also recommend restriction of mobilization even in absence of an EVD.

#### 3.2.4 Duration of the restriction of mobilization

Among those respondents that recommended restriction of the mobilization (24 physicians), the average duration of the restriction varied widely ranging between 1 and 21 days. The most common recommendations were to restrict mobilization for 7–10 days irrespective of the patients’ condition (seven respondents, 24.1%), until the aneurysm could be secured (five respondents, 17.2%) or until the EVD could be removed (two respondents, 6.8%). For two respondents (6.8%), the duration of the restriction of mobilization depended on the clinical scenario i.e., the origin of the SAH (aneurysmatic or idiopathic), status of the ruptured aneurysm (secured or not) if present and the presence of an EVD.

### 3.3 Restriction of HOB positioning

**Fig. 2:**
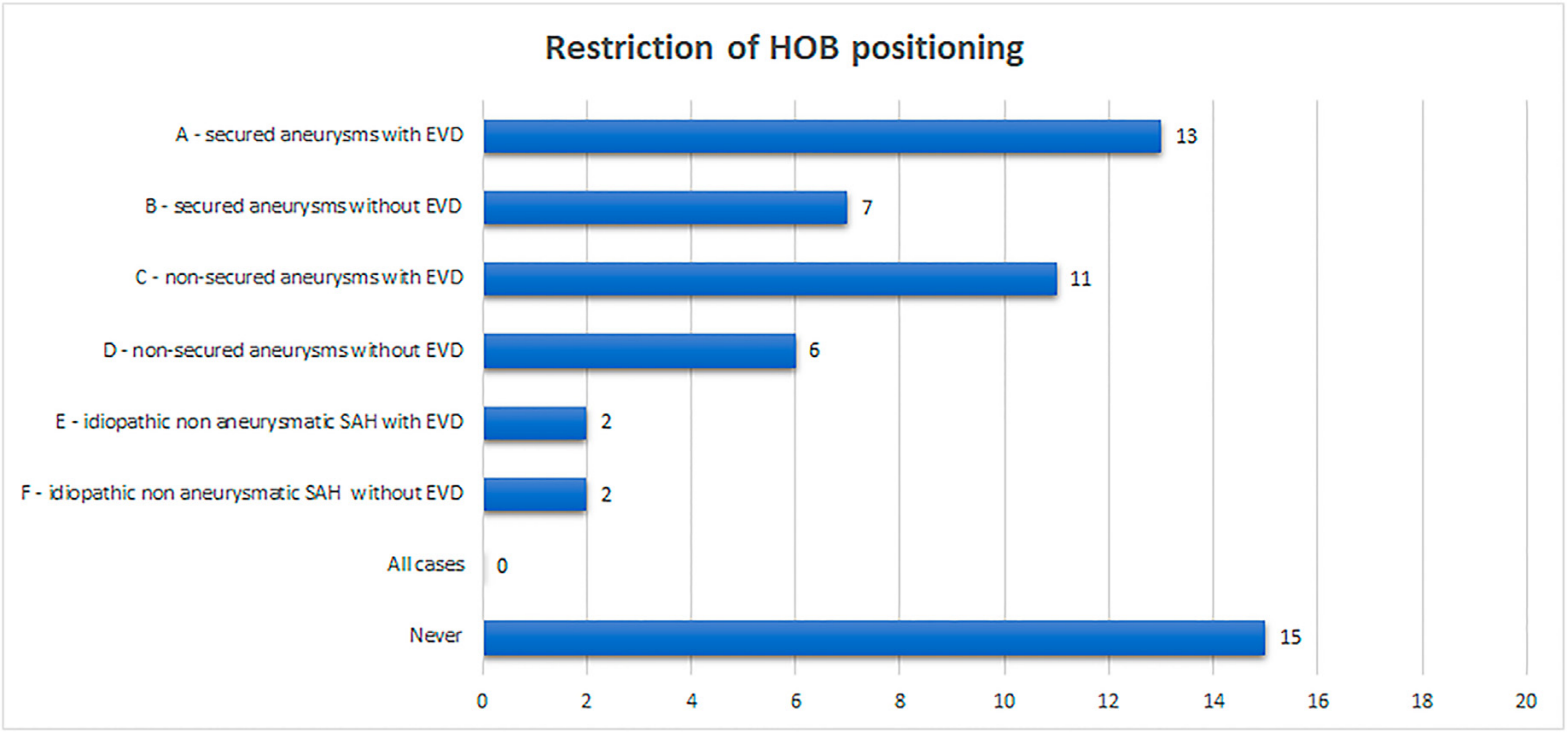
Differences in the recommendation for restriction of HOB positioning between different scenarios were detected (n = respondents).

Like the surveys’ findings on the restriction of mobilization after aSAH, differences in the recommendation for restriction of HOB positioning between different scenarios were detected (Fig. 2).

Most respondents (15, 51.7%) did not determine restriction of HOB positioning. Among those physicians that would recommend restriction of HOB positioning, respondents could select more than one answer for question 6: The most frequent combination of answers was to recommend restriction of HOB positioning for those patients that required the placement of an EVD (four respondents, 13.8%) and to restrict HOB positioning to patients with aSAH irrespective of the status of the aneurysm or the need of an EVD (four respondents, 13.8%).

#### 3.3.1 Secured aneurysms

For secured aneurysms, thirteen (44.8%) respondents would recommend restriction of HOB positioning for those patients that required EVD placement. However, for patients with secured aneurysm but without an EVD, the same restriction was only recommended by seven (24.1%) respondents.

#### 3.3.2 Non-secured aneurysms

For non-secured aneurysms, the rates of recommendation for restriction of HOB positioning were 37.9% (eleven respondents) and 20.7% (six respondents), respectively, depending on the presence or absence of an EVD.

#### 3.3.3 Idiopathic non-aneurysmatic SAH

For idiopathic non aSAH, restriction of HOB positioning was recommended by the same number of respondents for patients with or without an EVD, two (6.9%) in each case.

#### 3.3.4 Duration of the restriction of HOB positioning

The average duration of restriction of HOB positioning ranged between 3 and 14 days. Three respondents (10.3%) stated that it would be limited to the duration of the use of an EVD, and three respondents (10.3%) would recommend restriction of HOB positioning for 10–14 days for secured aneurysms independent of the presence of an EVD.

### 3.4 Reasons for restriction mobilization or HOB positioning

Respondents answered to question 8 with a brief text that can be summarized as follows:

For four respondents (13.7%), the only reason to recommend restriction of mobilization was to accomplish a protocol or “hospital customs”. A relationship between active mobilization and EVD-related complications or rebleeding was suspected by seven (24.1%) and four (13.8%) respondents, respectively. For seven respondents (24.1%), active mobilization was also associated with higher risks for vasospasm or delayed cerebral ischemia. Curiously, two respondents (6.9%) recognized that their local recommendations were given by intensive care physicians, but that they as neurosurgeons would not agree with the recommended restrictions. Finally, one respondent (3.4%) did not recommend restriction of mobilization because active mobilization could enhance recovery of cerebral autoregulation.

## 4 Discussion

Our survey study highlights for the first time, how heterogeneous the current local practice for mobilization/HOB positioning after aSAH is, reflecting the lack of any concrete evidence. The main findings of our survey were: (1) The average duration of the restriction of mobilization varied widely ranging between 1 and 21 days. (2) The average duration of restriction of HOB positioning ranged between 3 and 14 days. (3) Non-secured aneurysms and the presence of an EVD seemed to be the main reasons to recommend restricted mobilization and restriction of elevation of HOB.

One of the major barriers to early mobilization has been the fear of adverse effects such as rebleeding, the development of cerebral vasospasm and the increased risk of secondary brain damage due to hemodynamic changes in cerebral circulation (Osgood, 2021). In patients with an EVD, especially the risk of CSF over drainage has kept patients immobilized. These complications are important since they may correlate with worse outcome (Karic et al., 2017). Unfortunately, there is no consensus regarding body positioning and mobilization in patients with aSAH and has not been part of previous guidelines (Connolly et al., 2012). In addition, there is no conclusive large cohort study addressing in detail the impact of early mobilization on cerebral vasospasm leading to DCI and/or DND, the most feared secondary complications of aSAH.

Early microcirculatory hemodynamic changes following aSAH have been demonstrated by using digital subtraction angiography (DSA) and shown to correlate with functional outcome (Wen et al., 2022). Furthermore, impaired autoregulation following poor grade aSAH has been demonstrated and was correlated with low CBF, without, however, leading to higher rates of DCI in affected patients (Johnson et al., 2016). Although, these changes may occur and may render the injured brain vulnerable to secondary insults, there is no evidence that early mobilization contributes to more complications. On the contrary, data on early mobilization after aSAH remain generally scarce even though early rehabilitation after has been suggested (Saciri and Kos, 2002). In a retrospective single-center study on 25 patients with aSAH, functional training and therapeutic exercise in upright positions were initiated 3.2 days after the hemorrhage and were considered to be feasible and safe (Olkowski et al., 2013). Similarly, no increased complications could be observed in a prospective single-center study on 94 patients with aSAH that received stepwise mobilization as early as one day after aneurysm repair, and the risk of severe cerebral vasospasm was even found to be reduced compared to controls (Karic et al., 2017). For 17 aSAH patients with EVDs, nurse-driven mobilization 4.9 days after admission to the ICU was deemed to be feasible and safe as well, furthermore resulting in improved discharge disposition compared to immobilized patients in another single-center prospective study (Young et al., 2019). Home discharge could also be achieved more often in 13 patients with mobilization 4.2 days after SAH compared to delayed mobilized patients with additional positive effects on the functional discharge status in a retrospective cohort study (Okamura et al., 2021). Recently, feasibility and safety of early mobilization after aSAH was again proposed in a larger single-center retrospective study on 56 patients with a span to first walking of 5 days after the insult, with implications for reduced antibiotic use and improved independence as well (Yokobatake et al., 2022). All together these studies indicate benefit of early mobilization and more importantly the lack of complications associated with this practice.

Our study has a couple of limitations. Firstly, note that the survey was distributed to the staff neurosurgeons experienced in the management of aSAH. We did not ask who is responsible for aSAH management (neurosurgeon or intensivist) at each center. Secondly, the survey was distributed to total 316 neurosurgeons (206 in the Vascular section and 110 in the Trauma & Critical Care section). Unfortunately, the response rate was quite low even after the second reminder. However, the results indicate the huge variability and no evidence based practice.

## 5 Conclusion

Our current study demonstrates the wide variations of practice as well as replicate the idea that positioning regimens are largely based on traditions and fears at individual institutions. The current evidence does not support the idea of increased risk of complications associated with early mobilization, in the contrary this might be beneficial. **Large prospective studies and/or RCTs involving different parts of the world are needed to establish scientific evidence of the impact of early mobilization on the outcome of patients with aSAH.**

## Funding

The 10.13039/100008723Finnish Medical Foundation (IH), The 10.13039/501100004212Päivikki and Sakari Sohlberg Foundation (IH), The 10.13039/501100007417Paulo Foundation (IH), The 10.13039/501100003125Finnish Cultural Foundation (IH), The 10.13039/501100008590Swedish Stroke Association (LT), Swedish State Support for Clinical Research (ALF; #Lio-925101) (LT), 10.13039/501100009252SciLifeLab/KAW (ER), Wallenberg Clinical Fellow (ER), Beijerstiftelsen (ER), Swedish government and the County Councils (ER), The ALF agreement (ER).

## Ethical approval

Since this was a survey-based study, no further institutional ethical clearance was required.

## Declaration of competing interest

The authors declare the following financial interests/personal relationships which may be considered as potential competing interests:Iftakher Hossain reports financial support was provided by The 10.13039/100008723Finnish Medical Foundation, The 10.13039/501100004212Päivikki and Sakari Sohlberg Foundation, The 10.13039/501100007417Paulo Foundation, The 10.13039/501100003125Finnish Cultural Foundation. Lovisa Tobiesson reports financial support was provided by The 10.13039/501100008590Swedish Stroke Association, Swedish State Support for Clinical Research. Elham Rostami reports financial support was provided by Wallenberg Clinical Fellow, Swedish government and the County Councils, The ALF agreement.
