# Supplementary material for: Huge variability in restrictions of mobilization for patients with aneurysmal subarachnoid hemorrhage - A European survey of practice
Source: Brain Spine. 2023 Mar 21;3:101731. doi: 10.1016/j.bas.2023.101731 (PMC10293289; doi:10.1016/j.bas.2023.101731)
Supplement: Multimedia component 1 [file mmc1.docx]

Appendix A

1. Where are you based?

2. The name of your city and neurocenter / hospital

3. In your practice, is there a protocol in place for patients presenting with SAH?

Yes

No

4. In which of the following scenarios would you apply restriction of mobilization?

A - secured aneurysms with EVD

B - secured aneurysms without EVD

C - non-secured aneurysms with EVD

D - non-secured aneurysms without EVD

E – idiopathic non-aneurysmatic SAH with EVD

F - idiopathic non-aneurysmatic SAH without EVD

None

5. If you made a selection in the previous question, what is the average time duration of restriction of mobilization in each case? (You can answer by writing the letter of the selection - the average duration, e.g. A - 7 - 10 days)

6. If applying restriction of mobilization, in which of the scenarios would you apply restrictions of HOB?

A - secured aneurysms with EVD

B - secured aneurysms without EVD

C - non-secured aneurysms with EVD

D - non-secured aneurysms without EVD

E - idiopathic non-aneurysmatic SAH with EVD

F - idiopathic non-aneurysmatic SAH without EVD

None

7. If you made a selection in the previous question, what is the average time duration of restriction of HOB in each case? (You can answer by writing the letter of the selection - the average duration, e.g. A - 7 - 10 days)

8. In case of restrictions of mobilization and/or HOB, please write down your rationale. If you mention literature or training or personal experience, please elaborate.
